# Incremental predictive value of device and echocardiographic parameters for atrial high-rate episodes: a prospective cohort study

**DOI:** 10.1186/s12872-026-05573-7

**Published:** 2026-02-10

**Authors:** Thanh Van Le, Sang Doan, Linh Ha Khanh Duong

**Affiliations:** 1 Cho Ray Hospital, Ho Chi Minh, Vietnam; 2 Eye Hospital of Ho Chi Minh City, Ho Chi Minh, Vietnam

**Keywords:** Atrial High-Rate episodes (AHRE), Permanent pacemaker, Paced p-wave duration, Diastolic wall strain, Subclinical atrial fibrillation

## Abstract

**Introduction:**

Atrial High-Rate Episodes (AHRE) detected by cardiac implantable electronic devices are associated with an increased risk of stroke and atrial fibrillation. This study aimed to evaluate the independent and synergistic predictive value of clinical, echocardiographic, and device-derived parameters for the onset of AHRE ≥ 30 s.

**Methods:**

In this prospective single-center cohort study, 165 patients undergoing *de novo* dual-chamber pacemaker implantation were enrolled. Baseline clinical characteristics, echocardiographic indices (including Diastolic Wall Strain [DWS]), biomarkers (NT-proBNP), and device parameters (Atrial Pacing Percentage [AP%] and Paced P-wave Duration [PPD]) were analyzed. The primary endpoint was the first occurrence of AHRE ≥ 30 s.

**Results:**

During a median follow-up of 10.5 months, the incidence of AHRE was 21.8% (*n* = 36). Multivariate Cox regression identified five independent predictors: PPD ≥ 160 ms (HR 4.12), AP% ≥ 50% (HR 3.05), DWS ≤ 0.34 (HR 2.75), Log-transformed NT-proBNP (HR 1.28), and Age (HR 1.24 per 5-year increase). A comprehensive model integrating these factors demonstrated superior discrimination (C-index 0.81) compared to a baseline clinical model (C-index 0.69) and achieved a significant net reclassification index (NRI) of 0.41.

**Conclusion:**

Intrinsic electrophysiological markers (prolonged PPD) and mechanical indices of left ventricular stiffness (low DWS) are powerful independent predictors of AHRE. Integrating these parameters significantly improves risk stratification, offering a practical tool for personalized monitoring and management strategies in pacemaker recipients.

## Background

 The rapid advancement of cardiovascular implantable electronic devices (CIEDs), including permanent pacemakers (PMs), has facilitated continuous, non-invasive cardiac rhythm monitoring [1, 2]. This capability has led to the identification of a new arrhythmia entity: Atrial High-Rate Episodes (AHRE) and Subclinical Atrial Fibrillation (SCAF). AHRE is defined as episodes of atrial tachyarrhythmias (atrial fibrillation, atrial flutter, or atrial tachycardia) where the atrial rate exceeds a programmed threshold, typically ≥ 175 or ≥ 190 beats/minute, lasting for a minimum duration (commonly ≥ 5 min) [3, 4].

The detection of AHRE carries profound prognostic significance. Studies have demonstrated that AHRE is not only a precursor to clinical atrial fibrillation (AF) (patients with AHRE face an almost 6-fold increased risk of developing clinical AF) but is also a powerful independent predictor of serious thromboembolic events [5–8]. Specifically, AHRE episodes lasting ≥ 30 s have been shown to significantly increase the risk of ischemic stroke or systemic embolism (SE), with a hazard ratio (HR) of 4.41 [6]. Furthermore, the presence of AHRE is also associated with an increased risk of all-cause mortality (RR 1.57) and cardiovascular mortality (RR 1.80) in CIED patients [9].

The literature has identified multiple factors linked to the appearance and progression of AHRE in pacemaker recipients, which can be categorized into clinical/structural and device-related groups. Clinical and structural factors include traditional risks like advanced age (≥ 65 years) and history of hypertension as independent risks for prolonged AHRE (> 6 h) [10]. Structurally, echocardiographic parameters reflecting atrial remodeling and left ventricular dysfunction are strong predictors. An enlarged left atrial (LA) dimension, particularly ≥ 40 mm, significantly increases the risk of prolonged AHRE [11]. Recent evidence has highlighted the importance of non-invasive electrocardiographic markers, such as P-wave peak time, in identifying patients at high risk for AHRE development [12]. A deeper analysis shows that indices reflecting myocardial fibrosis and left ventricular diastolic dysfunction, such as the left ventricular stiffness index (LVSI > 0.13) and diastolic wall strain (DWS < 0.34), are independent predictors for AF in dual-chamber (DDD) pacemaker recipients [13]. The presence of sick sinus syndrome (SSS) as the pacing indication also more than doubles the risk of developing AHRE, highlighting that damaged atrial substrate is a critical precondition [14].

Device parameters and intrinsic electrophysiological markers (EGM) provided by the pacemaker also offer detailed data. A high atrial pacing percentage (AP%), particularly ≥ 50%, is a powerful independent predictor for the development of AHRE/SCAF (HR 2.44; OR 3.48) [15]. This is thought to be because conventional atrial pacing itself can promote the dispersion of intra-atrial conduction, facilitating arrhythmia. This is supported by the finding that prolonged Paced P-wave Duration (PPD) (≥ 160 ms), intrinsically recorded by the device, has become a strong electrophysiological marker, with an Odds Ratio of 4.2 in predicting AHRE lasting over 24 h [16]. Additionally, prolonged paced QRS duration (QRSd ≥ 142 ms) is also related to prolonged AHRE [17], suggesting a complex pathogenetic link between pacing-induced ventricular dyssynchrony and secondary left atrial pressure elevation.

While AHRE is positively associated with an increased risk of stroke, this relationship is complex and frequently characterized by a temporal dissociation between subclinical arrhythmia episodes and the thromboembolic event. Stroke risk in CIED recipients likely reflects a multifaceted interplay between baseline clinical risk factors (as quantified by the CHA_2_DS_2_-VASc score), atrial cardiomyopathy, and the cumulative burden of subclinical arrhythmia [18–21]. Although short AHRE episodes (≥ 30 s) are associated with high stroke risk, the optimal duration threshold for initiating OAC remains unsettled, with clinical guidelines suggesting a threshold around 5.5 to 6 h for continuous AHRE [18, 19, 22]. Furthermore, large clinical trials (such as ARTESiA) suggest that the stroke reduction benefit from OAC only outweighs the bleeding risk when patients have a very high baseline stroke risk (CHA_2_DS_2_-VASc ≥ 4), even with prolonged AHRE [19]. For patients with intermediate duration AHRE (> 6 min to < 5.5 h) or intermediate CHA_2_DS_2_-VASc scores, the OAC decision becomes particularly difficult and non-uniform [18, 19, 23].

This study aims to address the current knowledge gap regarding the predictive value of factors for AHRE lasting ≥ 30 s. The primary objective is to analyze the independent and synergistic predictive value of clinical factors, imaging parameters (echocardiography), implantable device parameters (including AP%, PPD, and paced QRSd), and circulating biomarkers (such as NT-proBNP and hsTnI) for the onset of AHRE in patients with permanent pacemakers. By identifying the strongest predictors for AHRE, this research hopes to provide a more accurate clinical tool, supporting the individualization of monitoring strategies, early intervention, and anticoagulation treatment decisions, especially for the intermediate-risk patient group.

## Methods

### Study design and ethical aspects

#### Study design

The study was conducted as a Prospective Single-Center Cohort Study.

#### Ethical aspects and compliance

The entire study protocol strictly adhered to the ethical principles of the Declaration of Helsinki. The study was approved by the Institutional Ethics Committee of the University of Medicine and Pharmacy at Ho Chi Minh City (Decision No. 806/HDDD-DHYD) and registered on ClinicalTrials.gov with the identifier NCT06174506. Written Informed Consent was mandatory for all subjects before any data collection procedures were performed.

### Study population

The study enrolled adult patients undergoing primary implantation of a permanent dual-chamber pacemaker at the Department of Arrhythmia Treatment, Cho Ray Hospital, between December 2023 and May 2024. A total of 173 patients were initially enrolled. After applying exclusion criteria (primarily due to failure to meet the minimum 6-month follow-up), 165 patients were included in the final survival analysis.

#### Inclusion criteria

Inclusion criteria were:

- Age ≥ 18 years.
- Indication for a permanent dual-chamber (DDD) pacemaker according to current clinical guidelines, primarily for Sick Sinus Syndrome (SSS) or Atrioventricular Block (AVB).
- Patient consent and ability to adhere to the scheduled follow-up (and remote monitoring, if applicable).

#### Exclusion criteria

Patients with a history or evidence of prior atrial arrhythmias were excluded to ensure a homogenous cohort, focusing on the *De Novo* onset of AHRE. Exclusion criteria included:

- Documented evidence or history of symptomatic or subclinical Atrial Fibrillation (AF), Atrial Flutter, or Atrial Tachycardia (AT) prior to enrollment.
- Ongoing atrial arrhythmia at the time of enrollment.
- Pacemaker replacement or upgrade where the previous device recorded AHRE/AF.
- Severe valvular heart disease or valvular prosthesis.
- Left Ventricular Ejection Fraction (LVEF) < 50% or other complex cardiomyopathies.
- Pregnancy.

### Baseline data and clinical factor collection

Clinical data were collected at the first baseline visit after pacemaker implantation.

- Demographic and Baseline Conditions: Age, Sex, BMI, and history of conditions like Hypertension, Diabetes Mellitus, Smoking, and Chronic Kidney Disease (eGFR < 60 mL/min/1.73m^2^) were recorded.
- Stroke Risk Assessment: The CHA_2_DS_2_-VASc score was calculated for each patient.
- Pacing Indication: Patients were categorized by pacing indication, SSS versus AVB.

### Echocardiography assessment

Comprehensive transthoracic echocardiography was performed at baseline, adhering to ASE chamber quantification guidelines.

- Structural and Functional Parameters: Left Atrial dimension (LA dimension) and LVEF were measured.
- Diastolic Wall Strain (DWS): Diastolic Wall Strain (DWS) was calculated as a measure of left ventricular stiffness using M-mode or 2D echocardiography in the parasternal long-axis view. DWS was defined using the formula: DWS = (LVPWs - LVPWd) / LVPWs [24], where LVPWs and LVPWd represent the left ventricular posterior wall thickness at end-systole and end-diastole, respectively. A threshold of DWS ≤ 0.34 was used to classify left ventricular stiffness.

### Biomarkers collection and analysis

Blood samples were drawn at baseline: N-terminal pro–B-type Natriuretic Peptide (NT-proBNP) and high-sensitivity Troponin I (hsTnI) were measured using immunoassay methods.

### CIED parameters collection and processing

Device parameters were extracted from the device memory (via in-clinic interrogation) at baseline and during the first 3-month follow-up check. All measurements were performed by two independent observers to ensure accuracy. The pacemakers used in this study were primarily from Medtronic, Abbott and Biotronik.

- Atrial Pacing Percentage (AP%): The mean cumulative AP% was calculated over the first 3 months of follow-up.
- Paced P-wave Duration (PPD): PPD was measured from the Atrial Intracardiac Electrogram (A-EGM) during atrial pacing at a stable rate. PPD was manually measured using electronic calipers at a sweep speed of 50 mm/s with standard band-pass filtering (30–100 Hz) to clearly identify the interval from the atrial pacing spike to the end of the EGM signal. The binary threshold of PPD ≥ 160 ms was used as a marker for underlying abnormal intra-atrial conduction (IAB).
- Paced QRS Duration (Paced QRSd): Paced QRSd was measured from a surface 12-lead ECG during ventricular pacing. A threshold of QRSd ≥ 142 ms was used as a marker for pacing-induced ventricular dyssynchrony.

### Primary endpoint definition and follow-up

#### Primary endpoint definition

The primary endpoint was the time from implantation to the first occurrence of an AHRE lasting continuously ≥ 30 s. In this study, the dual-chamber pacemakers were uniformly programmed with an AHRE detection threshold of ≥ 175 beats per minute. AHRE was defined as episodes of atrial tachyarrhythmia (atrial fibrillation, flutter, or atrial tachycardia) with an atrial rate exceeding the programmed detection threshold.

#### Follow-up procedure

Patients were followed continuously via in-clinic device checks. Device memory data were downloaded at each check (at 3 months and subsequent visits) to ensure no AHRE episodes were missed. The minimum follow-up duration was 6 months or until the primary endpoint occurred. To ensure the accuracy of the AHRE ≥ 30 s endpoint and to minimize misclassification risk, all device-detected episodes were manually adjudicated using stored Intracardiac Electrograms (A-EGMs). This adjudication was performed by two independent cardiologists blinded to baseline clinical data. Only episodes showing clear evidence of atrial tachyarrhythmia lasting continuously for ≥ 30 s were included in the final analysis. Discrepancies were resolved by consensus involving a third senior reviewer.

### Statistical analysis

All statistical analyses were performed using R software version 4.5.1.

#### Survival analysis and Cox regression models

- Survival Analysis: Kaplan-Meier curves were used to describe the cumulative probability of remaining AHRE-free over time.
- Cox Regression Model: The Cox Proportional Hazards Model was employed to determine independent Hazard Ratios (HR) for the primary endpoint.
  - Multivariable Analysis: Variables significant in univariable analysis (*P* < 0.05), along with clinically relevant confounders (Age, CHA_2_DS_2_-VASc), were included in the multivariable model.

#### Model performance and incremental value assessment

The incremental predictive value of the echocardiographic and device-derived electrophysiological markers for AHRE prediction was assessed.

**Fig. 1:**
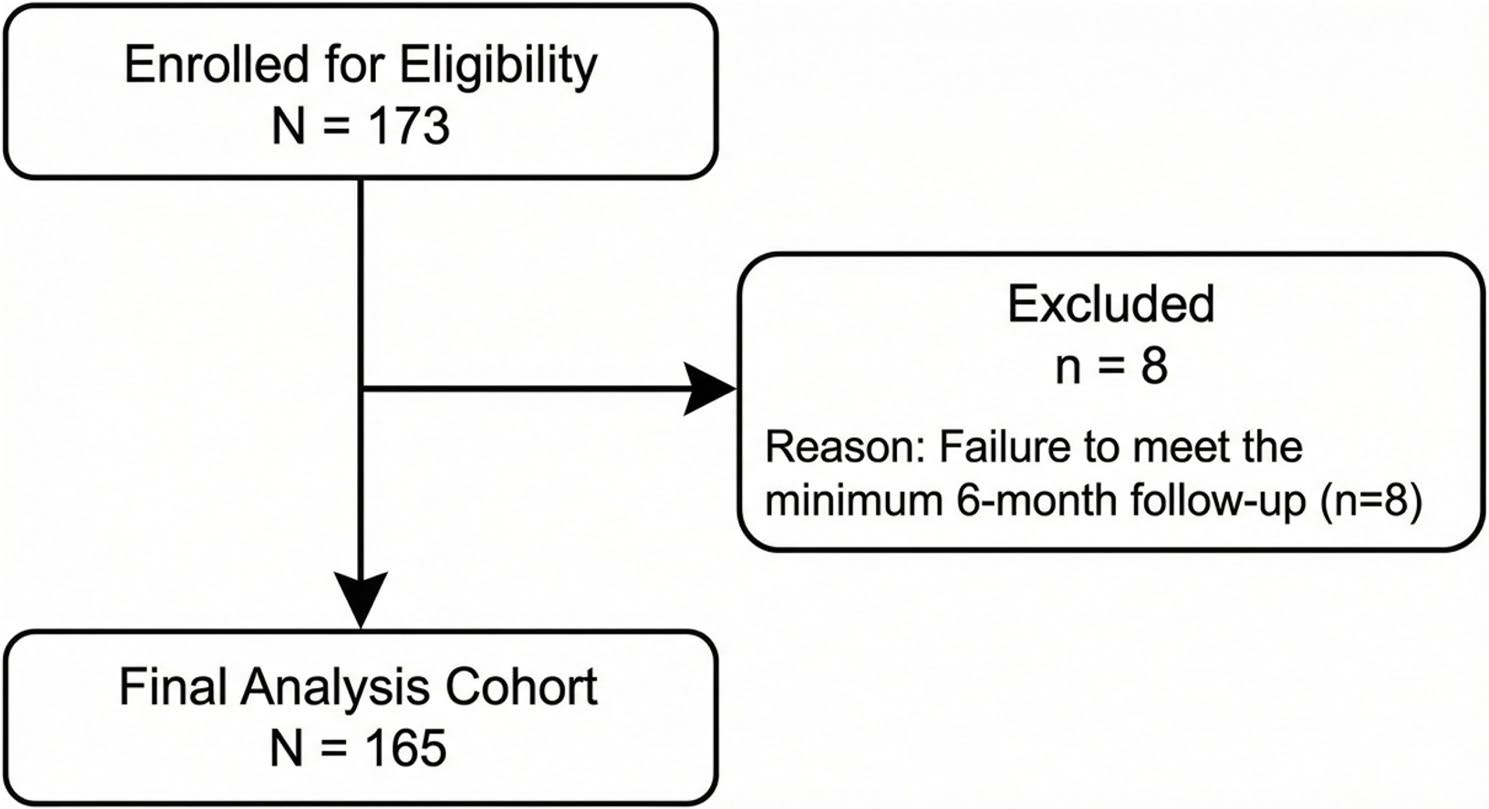
CONSORT flow diagram of the study population

- Discrimination: Model discrimination was evaluated using Harrell’s C-index. Three predictive models were compared:
  - Baseline Model (M1): Included Age, CHA2DS2-VASc, and Left Atrial dimension (LA).
  - Intermediate Model (M2): M1 + DWS + NT-proBNP.
  - Comprehensive Model (M3): M2 + PPD + AP%.
- Reclassification: The Net Reclassification Index (NRI) was used to quantify how much the inclusion of echocardiographic and device-derived markers (Model M3) improved the categorization of patients compared to the clinical model (M1). Patients were stratified into three risk categories based on their predicted probability of AHRE: Low (< 15%), Intermediate (15–30%), and High (> 30%) risk. Upward reclassification was defined as an event-positive patient moving to a higher risk category, while downward reclassification was defined as an event-negative patient moving to a lower risk category. NRI results were reported separately for event and non-event patients to highlight where the model provided the most significant gains in accuracy (Fig. 1).

## Results

### Baseline characteristics of the study population

A total of 173 adult patients undergoing de novo dual-chamber pacemaker (PM) implantation were initially recruited according to defined criteria. After applying exclusion criteria (primarily due to failure to meet the minimum 6-month follow-up time), 8 patients were excluded from the final survival analysis (Figure 1). The final analysis was conducted on 165 patients, comprising 60 males (36.4%) and 105 females (63.6%). The median follow-up time was 10.5 (IQR: 8.0–14.0) months. Baseline clinical, echocardiographic, and biomarker characteristics are detailed in Table 1, categorized by patients who developed AHRE (AHRE-Positive) and those who did not (AHRE-Negative). Baseline characteristics showed that the AHRE-Positive group was significantly older, presented as a median of 76.0 (IQR: 69.0–81.0) years compared to 70.0 (IQR: 60.0–77.0) years in the Negative group (*P* < 0.001). The proportion of patients with Sick Sinus Syndrome (SSS) as the indication for implantation was higher in the AHRE-Positive group (75.0% vs. 48.8%; *P* = 0.004). Biomarkers reflecting cardiac remodeling and more severe subclinical left ventricular dysfunction were significantly higher in the AHRE-Positive group, reflected by a substantially higher median NT-proBNP concentration (1522 [IQR: 677-4651] pg/mL vs. 296 [85-846] pg/mL; *P* < 0.001).

**Table 1:** Baseline characteristics of patients according to AHRE ≥ 30 s status

Variable	Total Cohort (*N* = 165)	AHRE-Negative (*N* = 129)	AHRE-Positive (*N* = 36)	*P*-value
Clinical Characteristics				
Age (years), Median (IQR)	71 (62–79)	70 (60–77)	76 (69–81)	< 0.001
Female, n (%)	75 (45.5)	54 (42.0)	21 (58.3)	0.045
SSS Indication, n (%)	90 (54.5)	63 (48.8)	27 (75.0)	0.004
CHA_2_DS_2_-VASc, Mean ± SD	3.5 ± 1.2	3.4 ± 1.1	3.9 ± 1.4	0.058
Hypertension, n (%)	120 (72.7)	95 (73.6)	25 (69.4)	0.621
Diabetes Mellitus, n (%)	48 (29.1)	35 (27.1)	13 (36.1)	0.342
Chronic Kidney Disease, n (%)	15 (9.1)	10 (7.8)	5 (13.9)	0.201
Echocardiographic Parameters				
Left Atrial Dimension (LA, mm), Mean ± SD	45.1 ± 6.2	43.5 ± 5.9	50.1 ± 7.5	< 0.001
DWS ≤ 0.34, n (%)	46 (27.9)	26 (20.2)	20 (55.6)	< 0.001
LVEF (%), Mean ± SD	61.5 ± 9.5	62.8 ± 8.9	57.1 ± 10.1	0.031
Biomarkers				
NT-proBNP (pg/mL), Median (IQR)	550 (127–1269)	296 (85–846)	1522 (677–4651)	< 0.001
hsTnI (pg/mL), Median (IQR)	15.8 (5.2–80.5)	10.1 (4.5–60.2)	55.0 (20.2–145.1)	< 0.001
Device Parameters				
AP% ≥ 50%, n (%)	25 (15.2)	6 (4.7)	19 (52.8)	< 0.001
PPD ≥ 160 ms, n (%)	20 (12.1)	4 (3.1)	16 (44.4)	< 0.001
Paced QRSd ≥ 142 ms, n (%)	35 (21.2)	21 (16.3)	14 (38.9)	0.007

*Abbreviations*: *SSS* Sick Sinus Syndrome, *LA* Left Atrial Dimension, *DWS* Diastolic Wall Strain, *LVEF* Left Ventricular Ejection Fraction, *AP%* Atrial Pacing Percentage, *PPD* Paced P-wave Duration

### Incidence of atrial High-Rate episodes (AHRE)

**Fig. 2:**
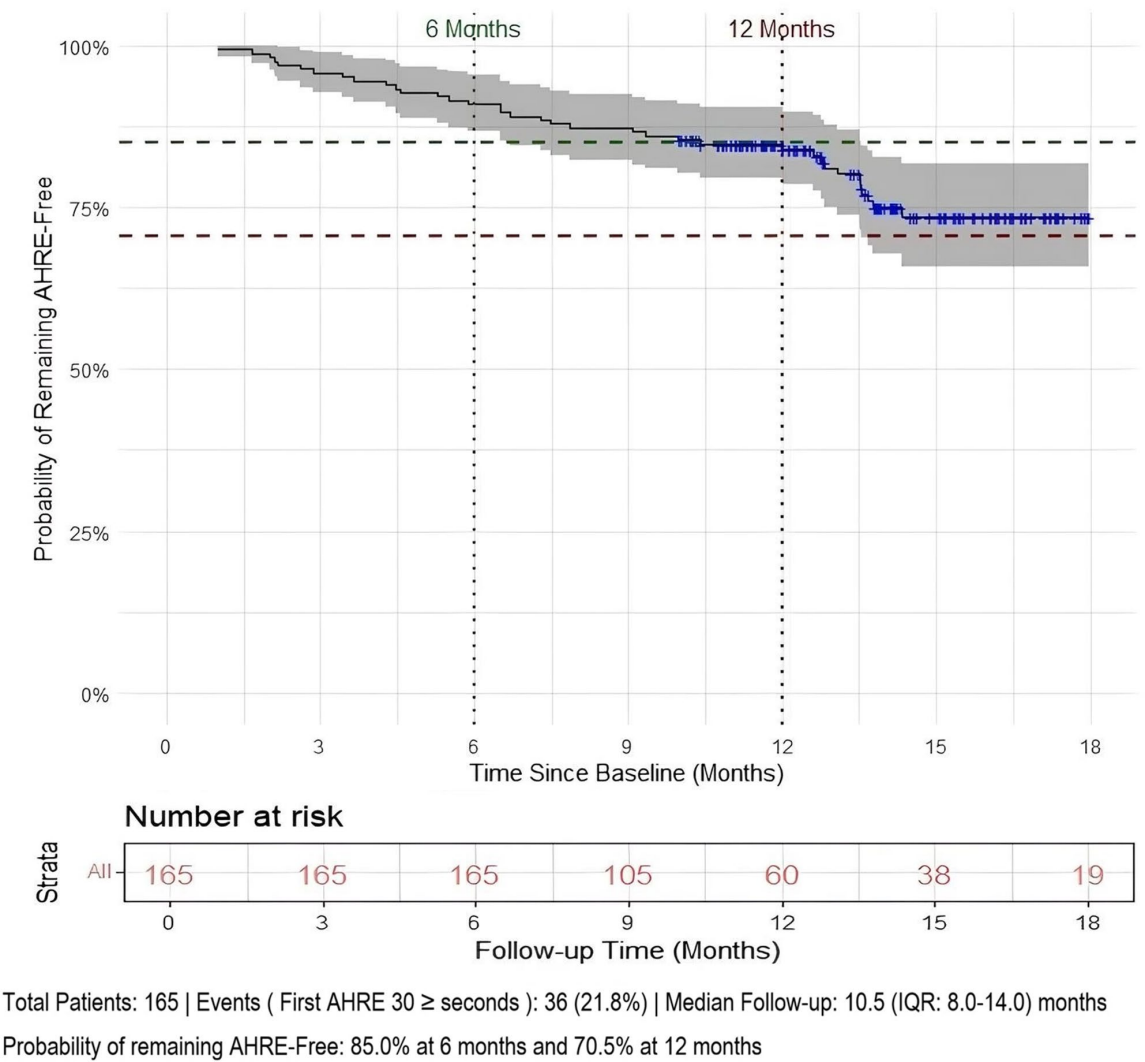
Kaplan-Meier curve showing the probability of remaining AHRE-free

During the median follow-up of 10.5 months, 36 patients (21.8%) reached the primary endpoint, which was the occurrence of the first continuous Atrial High-Rate Episode (AHRE) lasting ≥ 30 s. Kaplan-Meier survival analysis demonstrated that the probability of remaining AHRE-free was 85.0% at 6 months and 70.5% at 12 months (Fig. 2).

The vertical axis represents the estimated probability of a patient remaining AHRE-free over time. The horizontal axis shows the time since the baseline visit in months. The solid blue line traces the Kaplan-Meier survival estimate, with the surrounding shaded grey region indicating the 95% confidence interval. Small blue crosses (+) along the curve represent censored observations (patients who finished follow-up or withdrew before an event). Vertical dotted lines are drawn at the 6-month and 12-month time points. Corresponding horizontal dashed lines indicate the point estimate probabilities at these key intervals: the green line marks an 85.0% probability of remaining AHRE-free at 6 months, and the red line marks a 70.5% probability at 12 months. The “Number at risk” table below the x-axis displays the count of patients remaining in the study and event-free at each 3-month interval. An event was defined as the first AHRE lasting ≥ 30 s. The total study population consisted of 165 patients, with 36 (21.8%) experiencing an event. The median follow-up duration was 10.5 months (IQR: 8.0–14.0 months).

### Analysis of independent predictors for AHRE

#### Univariate Cox regression analysis

In univariate analysis, the variables including Age, SSS indication for PM implantation, NT-proBNP, hsTnI, Left Atrial Dimension (LA), DWS ≤ 0.34, AP% ≥ 50%, Paced QRSd ≥ 142 ms and PPD ≥ 160 ms all showed a significant association with the risk of developing AHRE ≥ 30 s.

#### Multivariate Cox regression model

**Fig. 3:**
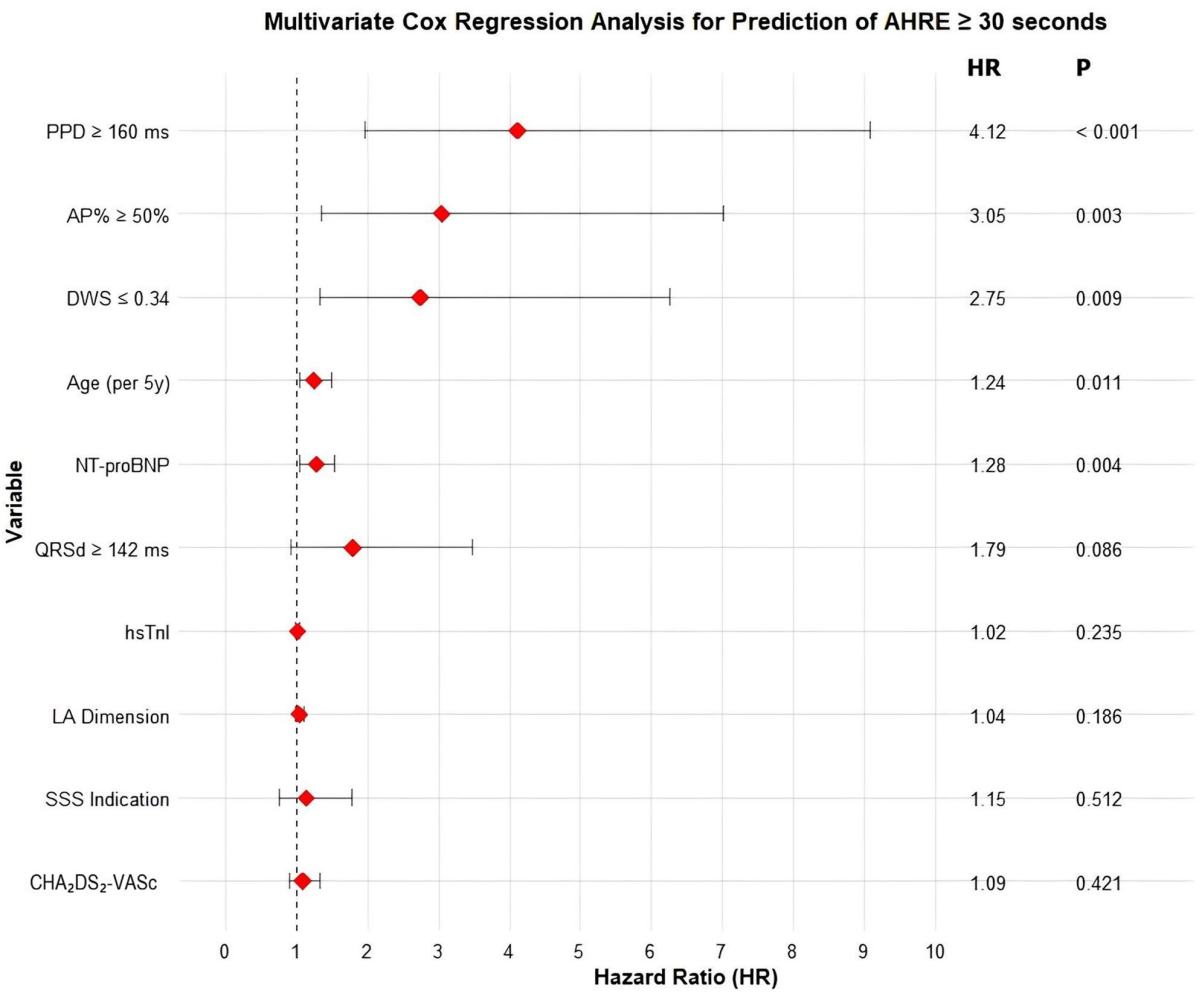
Forest Plot of Independent Predictors for Atrial High-Rate Episodes (AHRE) ≥ 30 s from Multivariate Cox Regression Analysis

Variables significant in the univariate analysis (*P* < 0.05) along with established confounding factors (Age, CHA_2_DS_2_-VASc) were entered into the stepwise multivariate Cox regression model. (Fig. 3)

This Forest plot displays the results of the multivariate Cox regression analysis for the independent predictors of AHRE ≥ 30 s. The red diamonds represent the point estimates of the Hazard Ratio (HR) for each variable. The horizontal lines through each diamond represent the 95% CI. A vertical dashed line is placed at HR = 1.0 (null effect). Variables are listed on the left, with corresponding HR and P-values displayed on the right.

After adjustment, only five factors were identified as strong and independent predictors for the onset of AHRE ≥ 30 s (Table 2). Although paced QRSd ≥ 142 ms was a significant factor in the univariate analysis, it did not reach statistical significance in the final multivariate Cox regression model (*p* = 0.086) and was therefore not identified as an independent predictor of AHRE.

**Table 2:** Independent predictors for AHRE ≥ 30 s (Multivariate Cox regression Analysis)

Variable	Hazard Ratio (HR)	95% CI	*P*-value
Device Parameters			
PPD ≥ 160 ms	4.12	1.97–9.08	< 0.001
AP% ≥ 50%	3.05	1.35–7.02	0.003
Structural/Biomarkers			
DWS ≤ 0.34	2.75	1.33–6.27	0.009
NT-proBNP (Log-transformed)	1.28	1.05–1.54	0.004
Clinical Factors			
Age (per 5-year increase)	1.24	1.04–1.50	0.011

The multivariate analysis results indicate that PPD ≥ 160 ms was the strongest predictor (HR 4.12), followed by high Atrial Pacing Percentage (AP% ≥ 50%) and the marker for left ventricular diastolic dysfunction (DWS ≤ 0.34)

### Evaluation of incremental predictive value of the model

We evaluated the discriminatory power C-index and reclassification ability of the model by adding imaging and device markers to a basic clinical model.

The discriminatory ability C-index showed that the Basic Model (M1), which included traditional clinical factors (Age, CHA_2_DS_2_-VASc, LA dimension), achieved a C-index = 0.69 (95% CI: 0.61–0.77). The Improved Model (M2) which added DWS and NT-proBNP significantly improved discrimination, raising the C-index to 0.76 (95% CI: 0.69–0.83) (*P* < 0.001 vs. M1). The Comprehensive Model (M3), which further added two device markers PPD and AP%) to M2, achieved a C-index = 0.81 (95% CI: 0.75–0.87), demonstrating superior predictive capability (*P* = 0.002 vs. M2).

The reclassification ability showed that the inclusion of DWS, NT-proBNP, PPD, and AP% into the prediction model M3 vs. M1 yielded a significant NRI of 0.41 (*P* < 0.001). For patients who *experienced* the AHRE event (positive), Model M3 correctly reclassified 32.5% into a higher risk category (NRI event = 0.325). For patients who *did not experience* the AHRE event (negative), Model M3 correctly reclassified 8.5% into a lower risk category (NRI non-event = 0.085). These results confirm that the combination of imaging and device electrophysiological factors provides significant incremental predictive value, surpassing traditional clinical and echocardiographic scores, and can be utilized for personalized management strategies for this patient cohort.

## Discussion

This prospective single-center cohort study aimed to analyze the independent and synergistic predictive value of multi-domain risk markers - clinical, echocardiographic, biomarker, and device-derived electrophysiological parameters - for the first onset of AHRE lasting ≥ 30 s in patients receiving their first permanent dual-chamber pacemaker. Our key findings were threefold: The cumulative incidence of AHRE ≥ 30 s was 21.8% over a median follow-up of 10.5 months. Multivariate Cox regression identified five independent predictors: PPD ≥ 160 ms (HR 4.12), AP% ≥ 50% (HR 3.05), DWS ≤ 0.34 (HR 2.75), log-transformed NT-proBNP (HR 1.28), and age (per 5-year increase) (HR 1.24). Crucially, the comprehensive model (M3), which integrates all identified factors, demonstrated superior discrimination (C-index = 0.81) and significant reclassification (NRI = 0.41) compared to a baseline clinical model (C-index = 0.69).

Our results largely corroborate and significantly extend previous findings on the pathogenesis of AHRE [25–29]. The intrinsic electrophysiological markers derived directly from the CIED proved to be the most potent predictors in our cohort. Specifically, a PPD ≥ 160 ms was associated with a more than four-fold increased risk of AHRE (HR 4.12). This finding is highly consistent with prior literature identifying prolonged PPD as a robust marker for underlying IAB [16], which reflects a pathologically remodeled atrial substrate highly susceptible to re-entry arrhythmias. Our results align with the growing body of evidence suggesting that delayed atrial conduction—whether measured via internal electrograms or surface electrocardiography—is a critical prognostic indicator. For instance, recent studies have demonstrated that other P-wave indices, such as P-wave peak time (PWPT) in leads V1 and D2, also possess high prognostic value in predicting the onset of AHRE [12]. Collectively, these data emphasize that assessing atrial electrical activity is crucial for accurate risk stratification in CIED recipients. Similarly, the predictive value of a high AP% ≥ 50% (HR 3.05) confirms that high pacing burden acts as an arrhythmogenic trigger in susceptible hearts. Furthermore, our study highlights the critical role of subclinical left ventricular dysfunction and systemic congestion. The inclusion of DWS ≤ 0.34, a robust marker for increased left ventricular stiffness and diastolic dysfunction, and elevated NT-proBNP, a biomarker reflecting myocardial wall stress [30, 31], supports the hypothesis that elevated left ventricular filling pressures contribute to chronic left atrial pressure overload, structural remodeling, and electrical instability [32, 33]. The finding that DWS and NT-proBNP significantly improved the model (M2, C-index 0.76) confirms the importance of these structural changes, which are often overlooked by simple LA dimension measurement.

The independent predictive value of a high AP% ≥ 50% (HR 3.05) should be interpreted with caution. As noted by others, this association likely reflects confounding by indication, as patients requiring frequent atrial pacing typically have underlying atrial cardiomyopathy or SSS, which are inherent risk factors for AHRE [34]. Furthermore, AP% should be interpreted in conjunction with PPD ≥ 160 ms; while PPD represents the diseased electrical substrate (intra-atrial conduction block), high AP% may act as a pacing-induced trigger [16]. It is also important to recognize that AP% may paradoxically decrease as AHRE/AF burden increases and pacing is no longer required, reflecting disease progression rather than risk reduction [34]. Finally, alternative pacing strategies that optimize interatrial conduction may mitigate the risk associated with high pacing burdens.

These results support a multifactorial vulnerability model for AHRE onset, integrating electrical, mechanical, and systemic factors. The M3 model suggests that the risk for AHRE is determined by the synergy between electrical substrate vulnerability (PPD ≥ 160 ms), mechanical overload (DWS ≤ 0.34, NT-proBNP), and a pacing-induced trigger (AP% ≥ 50%). This comprehensive view explains the superior performance of M3 (C-index 0.81), demonstrating that clinical risk scores and basic structural metrics alone are insufficient. Integrating intrinsic electrical markers and left ventricular stiffness provides a more complete picture of the electrophysiological and hemodynamic vulnerability that drives AHRE onset.

It is important to acknowledge that while we utilized a sensitive threshold of AHRE ≥ 30 s for early detection, the isolated occurrence of such short episodes does not necessarily confer a “high-risk” thromboembolic status. Large trials like ASSERT [5] have demonstrated that stroke risk increases primarily with longer episodes (> 6 min or > 24 h). Therefore, short-duration AHRE should be interpreted as high risk only when accompanied by additional structural, mechanical, or biomarker abnormalities - such as low DWS and elevated NT-proBNP - which are suggestive of an underlying atrial cardiomyopathy. Our study highlights that this specific subgroup, characterized by multi-domain electrical and mechanical vulnerability, is the most likely to represent individuals at truly elevated risk who require closer clinical attention.

The most profound clinical implication of this study is the significant incremental predictive value achieved by integrating readily available data from the pacemaker device and focused testing, validated by the high NRI of 0.41. This M3 model provides a practical tool for personalized risk stratification at the time of pacemaker check. Identifying high-risk patients (M3 correctly reclassified 32.5% of event-positive patients into a higher risk category) early allows for tailored management strategies, such as intensified device monitoring or early consideration of oral anticoagulation. This is particularly crucial for the intermediate-risk group where anticoagulation decisions are complex. Furthermore, the strong HR for PPD ≥ 160 ms emphasizes the importance of routinely extracting and analyzing the Atrial Intracardiac Electrogram (A-EGM) data during device interrogation.

The findings of this study have significant implications for the clinical management of dual-chamber pacemaker recipients. First, we recommend that PPD measurement be incorporated routinely into standard pacemaker interrogations. Given that PPD is the strongest independent predictor in our cohort (HR 4.12) and is easily extracted from the device’s intracardiac electrogram (A-EGM), it serves as a high-yield, non-invasive marker for identifying patients with a diseased atrial substrate. Second, the evaluation of DWS via baseline echocardiography provides a powerful tool for the early detection of AHRE risk. By identifying mechanical ventricular stiffness before advanced structural remodeling occurs, DWS allows for the early stratification of patients who may benefit from more frequent monitoring or intensified diagnostic screening. Integrating these readily available electrical and mechanical parameters into a comprehensive risk model allows clinicians to move toward a more personalized approach in managing subclinical atrial fibrillation.

Despite the strengths of this study - including its prospective cohort design, focus on the prognostically relevant AHRE ≥ 30 s endpoint, and the use of multi-modal data validated by advanced statistical metrics - several limitations must be acknowledged. First, the single-center nature of the investigation may restrict the generalizability of the findings to broader or more geographically diverse populations. Second, while the cohort of 165 patients provided sufficient power for the primary multivariate analysis, the sample size remains modest. Third, the median follow-up of 10.5 months is relatively short compared to larger landmark AHRE studies, potentially missing late-onset episodes or long-term clinical outcomes such as ischemic stroke and systemic embolism. Fourth, the comprehensive M3 predictive model developed in this study lacks external validation in an independent cohort, which is a necessary step to confirm its robustness and reliability before it can be widely implemented in clinical practice. Finally, although a sensitive threshold of AHRE ≥ 30 s was utilized for early detection, the isolated occurrence of such short episodes does not necessarily confer a high-risk thromboembolic status, as stroke risk has been shown to increase primarily with much longer episodes exceeding six minutes or 24 h. Therefore, these short-duration episodes should be interpreted as high risk only when accompanied by additional structural or mechanical abnormalities, such as low DWS and elevated NT-proBNP, which suggest underlying atrial cardiomyopathy.

## Conclusion

In conclusion, the combination of PPD ≥ 160 ms, AP% ≥ 50%, DWS ≤ 0.34, and NT-proBNP provides superior incremental value for predicting AHRE ≥ 30 s in dual-chamber pacemaker recipients. These markers should be integrated into clinical practice to achieve a more accurate and personalized risk stratification approach.

## Data Availability

The datasets used and/or analysed during the current study are available from the corresponding author on reasonable request.

## Abbreviations

AHRE: Atrial High-Rate Episodes
AP%: Atrial Pacing Percentage
AVB: Atrioventricular Block
CIEDs: Cardiovascular Implantable Electronic Devices
DDD: Dual-chamber (pacemaker)
DWS: Diastolic Wall Strain
eGFR: Estimated Glomerular Filtration Rate
HR: Hazard Ratio
hsTnI: High-sensitivity Troponin I
IAB: Intra-atrial Conduction Block
IQR: Interquartile Range
LA: Left Atrial
LVPWs: Left Ventricular Posterior Wall thickness at end-systole
LVPWd: Left Ventricular Posterior Wall thickness at end-diastole
LVEF: Left Ventricular Ejection Fraction
NRI: Net Reclassification Index
NT-proBNP: N-terminal pro–B-type Natriuretic Peptide
OAC: Oral Anticoagulation
PM: Pacemaker
PPD: Paced P-wave Duration
QRSd: Paced QRS duration
SCAF: Subclinical Atrial Fibrillation
SSS: Sick Sinus Syndrome
